# What is your diagnosis?

**DOI:** 10.4274/jtgga.galenos.2018.2018.0149

**Published:** 2019-05-28

**Authors:** Selçuk Sarıkaya, Emre Huri

**Affiliations:** 1 Clinic of Urology, University of Health Sciences, Gülhane Research and Training Hospital, Ankara, Turkey; 2 Department of Urology, Hacettepe University Faculty of Medicine, Ankara, Turkey

A 53-year-old female patient was admitted to Hacettepe University Faculty of Medicine, Department of Urology outpatient clinic with dysuria, swelling in the left inguinal region, and severe urinary incontinence symptoms. Informed consent was gained from the patient before the study. The patient had stress-type urinary incontinence and a history of midurethral sling surgery 10 years ago due to her symptoms. Incontinence symptoms reduced in the first three postoperative months but there was recurrence afterwards. The patient had hypertension but no other comorbidities. According to the physical examination, there was grade I-II cystocele and vaginal atrophy. The Bonney stress test was positive and there was erosion in the left inguinal region due to the previous sling operation. Ultrasonography revealed abscess formation and mesh erosion in the left inguinal region. The same findings were observed with computed tomography. Afterwards, cystometry was performed; the maximum vesical capacity was 637 mL and the maximum vesical pressure was 33 cm H2O. Simultaneously, cystography revealed urine leakage while standing and there were no trabeculations. The uroflowmetry result was normal and there was no residual volume. The abscess formation was thought to be as a result of the mesh inflammation. Mesh excision, abscess drainage, and fascial transobturator sling operation was planned for the patient. During the operation, an approximately 4 cm-diameter mass due to mesh inflammation was excised from the left inguinal region, reaching to the retropubic area (Figure 1).

## 

### Answer

Stress urinary incontinence is a commonly seen pathologic entity with a rate of 13% among women aged 19-44 years, and 22% among women aged 45-64 years (1,2). Unfortunately it is an underdiagnosed and underreported medical problem. SUI can be assessed with physical examination, leak abdominal pressure point, and some other tests. Patients must undergo basic evaluation with a voiding diary, cotton swab test, cough stress test, cystoscopy, post-voiding residual volume, and urodynamic studies. There are many treatment methods that are used for the treatment of patients with SUI. Basically, these methods can be divided into surgical and nonsurgical modalities. Duloxetine is a recent treatment choice as a medical treatment option and studies have shown its positive effects in treatment (3). Physical exercise also has positive effects and must be considered as a treatment option. Apart from these treatment modalities, surgery is also widely used in the treatment of SUI (4).  Pubovaginal sling is a commonly used surgical procedure because it has many advantages. This procedure has an excellent overall success and it is a good option with longer curative rates (5). Midurethral slings have been used more often than pubovaginal slings recently with good success rates because it has become the gold standard for the treatment of SUI (5,6). Despite the positive outcomes, sometimes there are complications regarding this procedure. Infection and abscess formation would be seen as serious complications. Sometimes these complications can be corrected via excision of mesh.

## Figures and Tables

**Figure 1:**
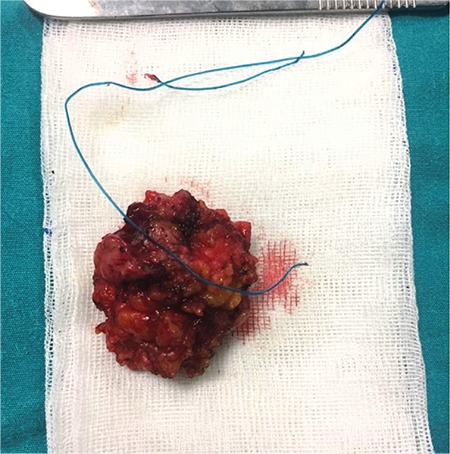
Macroscopic view of the excised abscess formation
